# Characterizing Vascular Wall and Lumen Caliber in Eyes with Diabetic Retinopathy Based on Adaptive Optics Scanning Laser Ophthalmoscopy

**DOI:** 10.3390/diagnostics14182020

**Published:** 2024-09-12

**Authors:** Konstantina Sampani, Mircea Mujat, Ankit H. Patel, Chaerim Kang, Nicusor Iftimia, Irini Chatziralli, Jennifer K. Sun

**Affiliations:** 1 Beetham Eye Institute, Joslin Diabetes Center, Boston, MA 02115, USA; 2 Department of Medicine, Harvard Medical School, Boston, MA 02115, USA; 3 Physical Sciences, Inc., 20 New England Business Center, Andover, MA 01810, USA; 4 Program in Liberal Medical Education, Brown University, Providence, RI 02903, USA; 5 Division of Ophthalmology, Alpert Medical School, Brown University, Providence, RI 02903, USA; 6 2nd Department of Ophthalmology, University of Athens, 12462 Athens, Greece; 7 Department of Ophthalmology, Harvard Medical School, Boston, MA 02115, USA

**Keywords:** adaptive optics, vascular remodeling, scanning laser ophthalmoscopy, diabetic retinopathy, diabetic retinal disease

## Abstract

(200/200) Purpose: Our aim was to evaluate structural alterations of retinal arterioles due to type 1 diabetes (T1D) and/or diabetic retinopathy (DR) under AOSLO imaging. Methods: Each study eye underwent mydriasis and AOSLO imaging in a single-visit study. The instrument’s arrangement of four offset aperture images provided two orthogonal split-detector images and enabled isotropic analysis of the arteriolar boundaries. For each arteriole, we calculated the wall-to-lumen ratio (WLR), mean wall thickness, and luminal and external diameters. Results: In total, we enrolled 5 (20.8%) healthy control eyes and 19 eyes of patients with T1D. The DR distribution was: four (16.7%) no-DR, nine (37.5%%) mild or moderate nonproliferative DR (NPDR), and six (25%) severe NPDR or proliferative DR. Mean wall thickness increased significantly in eyes with T1D compared to healthy controls (*p* = 0.0006) and in eyes with more advanced DR (*p* = 0.0004). The WLR was significantly higher in eyes with T1D (*p* = 0.002) or more severe DR (*p* = 0.004). There was no significant relationship between T1D status or DR severity and any of the arteriolar diameters. Conclusions: In this preliminary study, there appeared to be increases in the WLR and mean wall thickness in eyes with T1D and more severe DR than in the controls and eyes with no/less severe DR. Future studies may further elucidate the relationship between the retinal arteriolar structure and physiologic alterations in DR.

## 1. Introduction

Diabetic retinopathy (DR) is the most common microvascular complication of diabetes mellitus and a leading cause of blindness among working-age adults [1,2,3]. Its pathogenesis involves pericytes loss, basement membrane thickening, endothelial proliferation and disruption, and Muller cell degeneration [4,5,6,7,8]. Chronic hyperglycemia and inflammation due to diabetes not only contribute to vascular remodeling but also affect the retinal autoregulation and rheological properties of the blood, leading to capillary occlusion, nonperfusion and retinal ischemia [9]. Diagnosis of diabetic retinal disease relies on clinical observations of lesions within the retina and can be aided by the synergetic use of multi-modal retinal imaging modalities that visualize the neural and vascular retina. Identifying imaging biomarkers capable of detecting retinal microvascular alterations and neurodegeneration at earlier stages in order to improve risk stratification for future disease worsening would improve clinical management and treatment strategies [10].

The wall-to-lumen ratio (WLR) of the vasculature is a promising early marker of retinal microvascular changes, serving as an in vivo, quantitative parameter for arterial remodeling [11]. Elevated WLR values indicate potential arterial wall thickening, luminal narrowing, or both, and may imply vascular stenosis. Previous research demonstrated that WLR is a predictive biomarker linked to tissue hypoxia, carotid stiffness, cardiovascular events, and end-organ damage [12,13,14].

Several studies have investigated the link between WLR and DR using various retinal imaging modalities. Scanning laser Doppler flowmetry (SLDF) has been broadly utilized to measure retinal microcirculation. A recent SLDF study on patients with type 1 diabetes (T1D) found that WLR peaked in individuals with diabetes lasting over 10 years but did not explore its correlation with different DR stages [15]. Adaptive optics scanning laser ophthalmoscopy (AOSLO) is another retinal imaging technique that enables visualization of the retinal microvascular structure and hemodynamics. AOSLO findings in patients with diabetes showed increased WLR in eyes with nonproliferative DR (NPDR) when compared to healthy controls. Other studies focusing on patients with type 2 diabetes (T2D) reported comparable WLR between the diabetes and control groups [16], while another cross-sectional study noted an increase in WLR as DR severity advanced [17].

AOSLO provides cellular-level resolution images of the retinal microcirculation and structures by correcting for ocular aberrations in real time [18,19,20]. Its application in patients with DR enables quantitative assessment of DR-induced remodeling in retinal blood vessels, offering valuable insights that can aid in diagnosis and disease monitoring. To that end, this exploratory study employs AOSLO imaging to characterize the vascular morphology of retinal arterioles across the DR spectrum.

## 2. Materials and Methods

### 2.1. Study Cohort

This was a cross-sectional single-visit study conducted at the Beetham Eye Institute of the Joslin Diabetes Center in Boston. The study protocol was approved by the Joslin Diabetes Center Institutional Review Board (IRB) and adhered to the tenets of the Declaration of Helsinki. IRB approval was obtained to review the electronic medical record data of the patients described herein. The enrolled participants were 18 years or older, with T1D across the full spectrum of DR as well as healthy controls. Exclusion criteria included pupillary miosis or inability to dilate, prior panretinal photocoagulation, non-diabetic retinal pathology, media opacities limiting the ability to acquire high-quality AOSLO images, and untreated hypertension.

### 2.2. Study Procedures

Each study eye underwent mydriasis, ultrawide fundus photography (UWF) (Optos, Dunfermline, Scotland, UK), axial length measurement with the IOLMaster (Carl Zeiss Meditec, Carlsbad, CA, USA), and AOSLO imaging (Physical Sciences Inc., Andover, AM, USA) by trained personnel (K.S.) in this single-visit study. Comprehensive dilated eye examination was performed and DR severity was graded by retina specialists according to the Early Treatment Diabetic Retinopathy Study (ETDRS) DR severity scale during clinical examination after reviewing retinal UWF color photographs. Moreover, UWF images were used to locate the arterioles of interest within 1 disc diameter (DD) from the optic disc margin in the superior nasal and inferior nasal peripapillary quadrants. All arterioles imaged with AOSLO were located nasal to the disc due to the instrument’s capability. The AOSLO imaging mode provides location and coordinates tracking within the retina allowing for a standardized imaging pattern that is consistently applied to all patients. Demographic information along with diabetes-related data were recorded in a standardized manner.

### 2.3. AOSLO Imaging System

The multimodal adaptive optics retinal imager (MAORI, Physical Sciences Inc., Andover, MA, USA) used for AOSLO imaging in this study has been previously described in detail [20,21]. MAORI consists of a scanning laser ophthalmoscope (SLO) and a spectral domain optical coherence tomography (SDOCT) channel, simultaneously acquired and assisted by AO for compensating the optical aberrations induced by imperfect ocular optics. The detection module in the AOSLO channel uses a fiber bundle to simultaneously acquire one confocal and four offset aperture [22] images which provide complementary information and enable isotropic imaging of retinal microstructures, such as the cellular details of the blood vessel wall, independent of their spatial orientation [20].

### 2.4. Vessel Segmentation and Morphological Analysis

The AOSLO image post-processing steps have also been previously described in detail [20]. Typically, 100–200 frames are acquired at a retinal location at a 30–40 frames/second rate. The best, undistorted images are aligned [23] and mean and standard deviation images are calculated for each stack. The motion of blood cells through capillaries generates intensity fluctuations and therefore large standard deviation values that are translated into motion contrast. The standard deviation image (STD) illustrates the location of the blood flow without the need of additional contrast agents (like fluorescein) and enables direct segmentation of the vessel lumen.

The four offset aperture images were used to calculate the magnitude of the phase gradient (MPG) image [20]. The maxima in MPG images highlight the edges and borders of the blood vessel wall due to the refractive index discontinuities at these locations characteristic of tissue structural variations.

**Figure 1:**
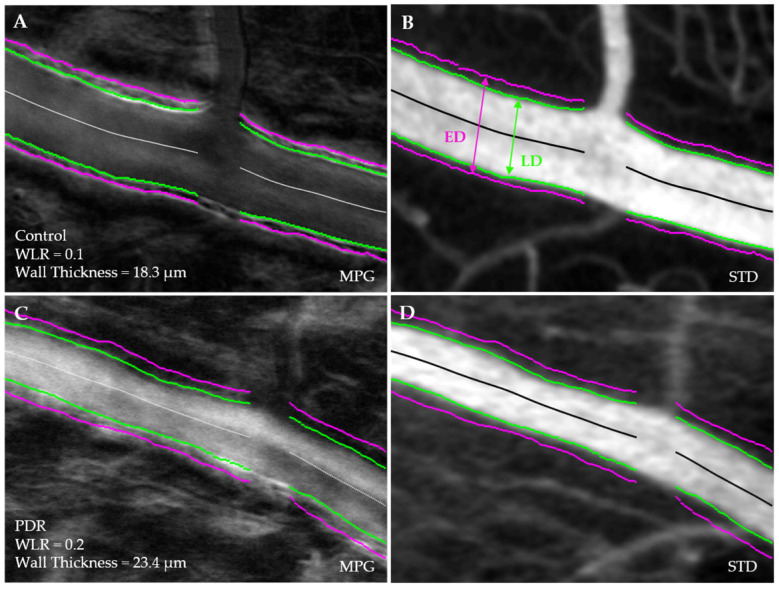
Magnitude of phase gradient (MPG) and standard deviation (STD) images of arterioles with delineated luminal (LD) (green) and external (ED) (magenta) boundaries. (**A**,**B**): Arteriole of a healthy control eye. (**C**,**D**): Arteriole of an eye with proliferative diabetic retinopathy (PDR).

An example of the simultaneous segmentation of the STD and MPG images is shown in Figure 1. The green curve illustrates the lumen as the boundary of the blood flow in the STD image as well as the inside edge of the vessel wall in the MPG image. The outside edge of the vessel wall is outlined by the magenta curve corresponding to a local maximum in the MPG images.

A custom-made Matlab program provides the initial segmentation of the MPG and STD images by localizing the maxima in the MPG and the flow border in STD. Fine-tuning can then be performed manually to correct for discontinuities and missed boundaries. In some instances, the walls of mural cells were highlighted by the Matlab code as the local maxima in the MPG images. Clearly, such edges were not representative of the external wall of the blood vessel and were creating discontinuities in the wall segmentation. They were erased manually, and the external wall edge was completed at those locations similarly to the snake-like segmentation procedures that keep edges continuous by balancing gradient forces with continuity requirements. Such automatic procedures will be implemented in future iterations of the segmentation algorithm.

The arteriolar parameters of interest that were measured at each arteriolar segment were the wall-to-lumen ratio (WLR), mean arterial wall thickness, luminal (LD) and external (ED) diameters.

### 2.5. Statistical Analysis

The segmentation of all the arterioles was performed by 2 trained graders, KS and MM. For agreement between graders, <10% area variation for each vessel and sub-region was ensured. All statistical analyses were completed using SAS statistical software (SAS, version 9.4; SAS Institute Inc., Cary, NC, USA). This study’s outcome measures were T1D presence and DR severity level and the predictor variables were the WLR, mean wall thickness, and luminal and external diameters. The Wilcoxon rank-sum test was used to test for significance in the comparison of the means between T1D and DR groups as well as for pairwise comparisons within the different DR severity levels. In this exploratory analysis, *p* values ≤ 0.05 were considered significant and *p* values ≤ 0.1 were considered trend-level.

## 3. Results

In total, nineteen patients were enrolled in the study. We imaged 24 study eyes with MAORI and segmented 110 arterioles within 1 DD from the optic disc margin. The participants’ ocular characteristics can be found in Table 1 and Table 2, respectively.

**Figure 2:**
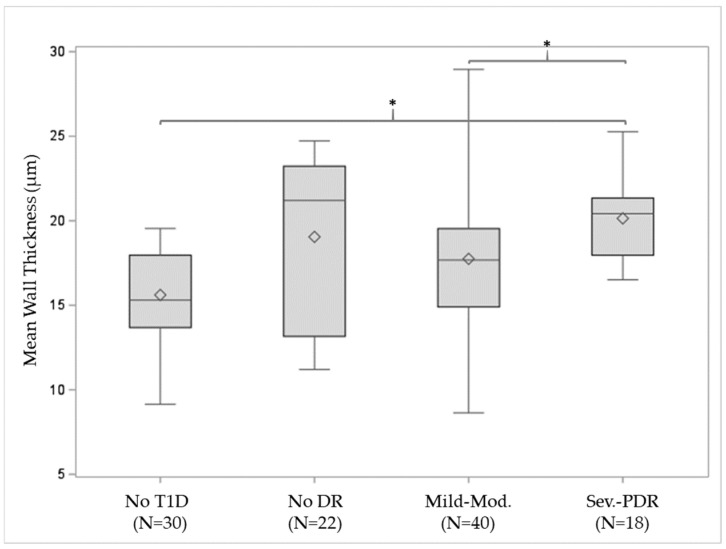
Box plot for the mean wall thickness against DR distribution. The bottom and top lines of the boxes correspond to first and third quartiles, respectively; the middle lines refer to the median values, the diamonds to the mean values, and the whiskers to the standard deviations. The nonparametric test showed significant greater mean wall thickness as DR severity advances (*p* = 0.0004). Pairwise comparisons within the DR severity levels showed that that severe NPDR–PDR group had significantly increased thickness as compared to the healthy controls (*p* < 0.001) and mild–moderate NPDR group (*p* = 0.03). * Statistical significance; T1D: type 1 diabetes; DR: diabetic retinopathy; PDR: proliferative DR.

Arteriolar structural measurements by participants’ T1D status can be found in Table 3. Mean wall thickness increased significantly in eyes with T1D when compared to the healthy controls (*p* = 0.0006), as can be seen in Table 3. Furthermore, the wall was found to be significantly thicker in eyes with more advanced DR (*p* = 0.0004), as shown in Figure 2 and Table 4. When using pairwise comparisons to test the inter-DR-group differences we found that severe NPDR–PDR group had significantly increased thickness compared to the healthy control (*p* < 0.001) and mild–moderate NPDR group (*p* = 0.03). None of the other pairwise comparisons met statistical significance.

**Figure 3:**
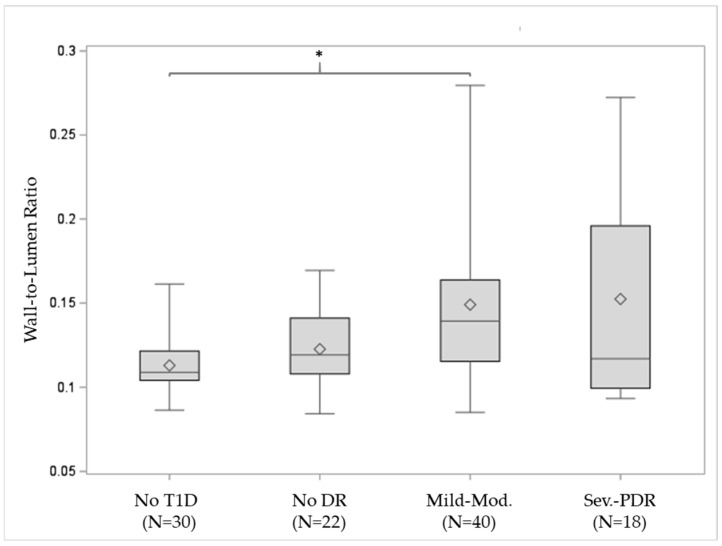
Box plot for the wall-to-lumen ratio (WLR) against DR distribution. The bottom and top lines of the boxes correspond to first and third quartiles, respectively; the middle lines refer to the median values, the diamonds to the mean values, and the whiskers to the standard deviations. The nonparametric test showed a significantly greater WLR as DR advances (*p* = 0.004). Pairwise comparisons within the DR severity levels revealed a significantly greater WLR in eyes with mild or moderate NPDR when compared with healthy eyes (*p* = 0.002). * Statistical significance; T1D: type 1 diabetes; DR: diabetic retinopathy; PDR: proliferative DR.

The wall-to-lumen ratio was found to be significantly greater in eyes with T1D when compared to healthy controls (*p* = 0.002), as shown in Table 3. When stratifying by the different severity levels of DR, the WLR remained significantly increased in eyes with more severe DR (*p* = 0.004) (Figure 3). Pairwise Wilcoxon comparisons demonstrated a significantly increased WLR in eyes with mild or moderate NPDR against healthy controls (*p* = 0.002). None of the other pairwise comparisons demonstrated any significance.

There was no significant relationship between T1D status or DR severity and either luminal or external arteriole diameters (T1D status: luminal *p* = 0.9; external *p* = 0.5, see Table 3; DR severity luminal *p* = 0.062; external *p* = 0.063).

## 4. Discussion

In this study, we segmented and characterized the structural characteristics of peripapillary retinal arterioles in eyes of healthy controls and patients with T1D by using AOSLO imaging. Our findings are consistent with greater arteriolar remodeling in eyes with T1D or advanced DR, in that the mean wall thickness and WLR were increased in the T1D group and advanced DR when compared to controls. We did not observe any significant relationship between T1D status or DR severity and either luminal or external arteriole diameters.

The retinal arterial wall integrity relies on multilayered smooth muscle cells that surround the artery and regulate the lumen’s diameter by contracting or relaxing according to the different metabolic demands [24], and the monolayer of endothelial cells that line the innermost layer of the artery and come in direct contact with blood flow [25]. Chronic exposure to hyperglycemia, hyperinsulinemia, and hyperlipidemia due to T1D compromises these vascular cells and causes dysfunction and loss of smooth muscle cells [26] and subsequent endothelial cell proliferation, dysfunction, and basement membrane thickening [27,28,29,30]. In human eyes with diabetes, prior AOSLO studies have visualized and quantified these structural arteriolar wall changes and showed greater loss of smooth muscle cells in eyes with subclinical DR when compared to healthy controls and also when DR is more advanced [31]. Wall thickness is a commonly used AOSLO imaging marker that reflects these cellular-level structural alterations due to diabetes and other systemic conditions [32]. Former AOSLO research has shown greater wall thickness in retinal arterioles and capillaries in eyes with minimal or no DR but most of these studies included small-diameter arterioles and mostly patients with type 2 diabetes [32,33,34]. Sapoznik et al. assessed only the small-diameter arterioles as a location more susceptible to early structural changes in diabetes (both T1D and T2D, no distinction) and they found that wall thickness and the WLR were increased in diabetes when compared to controls. In this study, we focused on large-diameter arterioles of eyes with T1D across the full spectrum of DR, including eyes with proliferative DR, and demonstrated that increased wall thickness is present in peripapillary arterioles of eyes with T1D or in eyes with more severe DR, implying that monitoring of arterial wall thickness may serve as surrogate for early detection and progression of DR.

Another common vascular remodeling marker in AOSLO research is the wall-to-lumen ratio, an in vivo parameter that has been associated with arteriolar damage. Higher retinal WLR has been linked with diabetes, hypertension, and other systemic parameters associated with diabetes management. Factors associated with a greater WLR include the thickening of the arterial wall, or narrowing of the lumen, or a combination of both. Meixner et al. showed that arterial hypertension and higher BMI are significantly associated with an increased retinal WLR due to arterial narrowing with unchanged wall thickness [13]. Dabrowska et al. found that among hypertensive patients, lumen narrowing and a greater WLR were significantly associated with cardiac damage [11]. Ueno et al. demonstrated that besides type 2 diabetes and hypertension, higher LDL blood levels are also significantly associated with greater WLR in eyes with PDR due to wall thickening and consequent narrowing of the lumen [17]. Recent AOSLO data of patients with diabetes found that the retinal WLR is also greater in both type 1 and 2 diabetes and more advanced DR stages in arterioles with diameter <50 μm [33]. Our findings showed an increased mean WLR in severe NPDR and PDR group, compared to healthy or no DR eyes, indicating that besides wall thickness, luminal narrowing may serve as a marker for advanced disease.

The WLR may be further utilized as a marker for investigating fluctuations of retinal blood flow in DR. Blood flow changes in the retina are linked with neurovascular coupling responses according to metabolic demands. Diabetes is a major contributor to the neurovascular coupling impairment. Multiple studies on DR have shown that neurodegeneration may precede vascular alterations [35,36,37]. Thus, developing sensitive, noninvasive imaging markers that detect neurovascular coupling changes early may further support clinical management of DR. In hypertension, the arteriolar response to flicker stimulant has been found to be diminished and negatively correlated with the WLR, implying changes in the retinal blood flow circulation. Prior animal studies in diabetes have shown that rats with diabetes had greater decreased blood flow than control rats and increases in vitreous vascular endothelial growth factor (VEGF) provoked significantly greater increases in blood flow [38,39,40]. In human diabetic retinas, researchers have observed a slowing of blood flow prior to the development of mild NPDR with subsequent increases as DR advances [38]. In this study, we showed that eyes of healthy controls and no DR had a lower WLR than the eyes with mild–moderate NPDR, indicating that these WLR findings may parallel DR blood flow alterations.

This study had some limitations. First, we imaged large arterioles within 1 DD from the optic disc margin and did not include small-diameter arterioles or capillaries that may be affected differently by diabetic metabolic effects. The smaller-diameter arterioles were not included in this study; hence, any conclusions about the vessel size and WLR in DR cannot be drawn. Retinal arterioles around the optic disc were identified first on colored fundus images and then imaged on AOSLO based on real-time image quality and the patient’s ability to fixate on the target. The imager followed a standardized path among the patients by imaging first the inferior nasal quadrant followed by the superior nasal quadrant within 1 DD from the optic disc margin. However, we acknowledge that we did not image all arterioles included in the peripapillary quadrants and this selection process may therefore be prone to selection bias. Nevertheless, the arterioles included in this study were located across the peripapillary area, and an attempt was made to select a representative sample of arteriolar branches. Future investigations should quantify the differences among the different diameter arterioles. Furthermore, we used quantification vessel markers that provide the wall and lumen dimensions without taking into consideration pathological findings across the borders of either the wall or the lumen, such as border discontinuity, hyper- or hypo-reflective signs, or other morphological lesions. Any ultrastructural vascular remodeling signs on AOSLO scans should be further evaluated and explored for association with diabetes and DR as well as a potential target for therapeutic molecule development in clinical trials for neurodegenerative changes in DR. Besides diabetes, hypertension is another systemic factor that contributes to vascular remodeling including the fine retinal arterioles [41,42]. In this cohort, 26.3% of the patients were receiving antihypertensive treatment. Notably, Harazny et al. showed that even within the patients under antihypertensive medication there is WLR variability due to poor blood pressure control and that poor control is associated with a higher retinal WLR. Future AOSLO studies may further quantify the impact of blood pressure control on diabetic vessel remodeling. Moreover, this study only enrolled patients with type 1 diabetes, excluding any patients with type 2 diabetes. Therefore, any conclusion for the comparison of the effect of type of diabetes on vascular wall reconstruction cannot be drawn. Lastly, our findings were drawn from a relatively small sample of study participants in a cross-sectional format, and future definitive, longitudinal studies will require larger and more diverse patient cohorts.

## 5. Conclusions

AOSLO imaging allowed for in-depth evaluation of the morphology of the retinal arterioles across the full spectrum of DR severity. In this preliminary study, there appeared to be increases in the wall-to-lumen ratio and mean wall thickness in eyes of patients with diabetes. Moreover, eyes with more advanced DR appeared to have a greater WLR and thicker mean wall thickness. If these findings are validated in larger, longitudinal cohorts, future studies will provide insight into correlations between retinal arteriolar structure and physiologic alterations in DR. These investigations should assess the impact of diabetes type and duration, arterial diameter, and systemic comorbidities on wall thickness and luminal diameter over time as well as investigate the relationship between arterial remodeling and neurovascular coupling impairment in eyes across the full spectrum of DR. As we transition into an era of individualizing patient care, it is crucial to develop reproducible markers that detect neurovascular subclinical and progression signs in DR and further allow individualized clinical intervention.

## Figures and Tables

**Table 1:** Participants’ demographic and T1D characteristics.

Participants Total N = 19	N (%) Mean ± SD
Age (years)	37.7 ± 8.1
Gender (Female)	10 (52.6)
Type 1 diabetes	14 (73.7)
T1D duration (years)	24.0 ± 8.2
Hemoglobin A1c (%)	7.4 ± 1.9

SD: standard deviation; T1D: type 1 diabetes.

**Table 2:** Participants’ ocular data.

DR Distribution	Eyes N (%)	Arterioles N (%)
Control	5 (20.8)	30 (27.3)
No DR	4 (16.7)	22 (20.0)
Mild–moderate NPDR	9 (37.5)	40 (36.4)
Severe NPDR–PDR	6 (25.0)	18 (16.4)
Total	24	110

NPDR: nonproliferative DR; PDR: proliferative DR.

**Table 3:** Vessels’ structural measurements between healthy controls and T1D.

Arteriolar Parameter N = 110	Controls IQR (Q1, Q3)	T1D IQR (Q1, Q3)	*p* Value
Wall-to-lumen ratio	0.02 (0.10, 0.12)	0.05 (0.11, 0.16)	0.002 *
Mean wall thickness (μm)	4.3 (13.7, 18.0)	6.0 (15.7, 21.7)	0.0006 *
Luminal diameter (μm)	28.0 (131.6, 159.6)	91.4 (91.8, 183.3)	0.907
External diameter (μm)	38.6 (159.0, 197.5)	99.6 (123.2, 222.7)	0.548

T1D: type 1 diabetes; IQR: interquartile range; * statistical significance; *p* values are calculated by nonparametric Wilcoxon tests.

**Table 4:** Vessels’ structural measurements between healthy controls and eyes with DR.

Arteriolar Parameter N = 110	Controls IQR (Q1, Q3)	No DR IQR (Q1, Q3)	Mild-Mod. NPDR IQR (Q1, Q3)	Severe—PDR IQR (Q1, Q3)
Wall-to-lumen ratio	0.02 (0.10, 0.12)	0.12 (0.11, 0.14)	0.05 (0.12, 0.16)	0.10 (0.10, 0.20)
Mean wall thickness (μm)	4.3 (13.7, 18.0)	10.07 (13.16, 23.23)	4.63 (14.91, 19.53)	3.3817.96, 21.34)
Luminal diameter (μm)	28.0 (131.6, 159.6)	127.20 (91.38, 218.58))	62.07 (91.40, 153.47)	79.63 (102.64, 182.27)
External diameter (μm)	38.6 (159.0, 197.5)	145.15 (115.24, 260.39)	63.92 (121.11, 185.02)	82.31 (140.31, 222.62)

T1D: type 1 diabetes; IQR: interquartile range; NPDR: nonproliferative diabetic retinopathy; PDR: proliferative diabetic retinopathy.

## Data Availability

The data presented in this study are available on request from the corresponding author. The data are not available due to privacy restriction.
